# Single-cell RNA-seq reveals a repair pattern in cystic lesions in steroid induced osteonecrosis of the femoral head

**DOI:** 10.3389/fimmu.2025.1626337

**Published:** 2025-09-01

**Authors:** Peng Peng, Jiaqing Tian, Kun Lin, Weihua Fang, Fangming Yao, Xiaoqiang Yang, Wenyuan Hou, Fangjun Xiao, Shun Lu, Wei He, Mincong He, Huan Xiao, Qiushi Wei

**Affiliations:** ^1^ Guangdong Provincial Second Hospital of Traditional Chinese Medicine, Guangzhou, China; ^2^ National Key Laboratory of Traditional Chinese Medicine Syndrome, Guangzhou, China; ^3^ The Fifth Clinical College of Guangzhou University of Chinese Medicine, Guangzhou, China; ^4^ Guangzhou University of Chinese Medicine, Guangzhou, China; ^5^ Guangdong Research Institute for Orthopedics and Traumatology of Chinese Medicine, Guangzhou, China; ^6^ Department of Orthopaedics, The Third Affiliated Hospital, Guangzhou University of Chinese Medicine, Guangzhou, China; ^7^ Department of Orthopedics, Bijie Traditional Chinese Medicine Hospital, Bijie, China

**Keywords:** steroid-induced osteonecrosis of the femoral head, single-cell RNA sequencing, chondrocyte, cystic lesions, osteogenic differentiation

## Abstract

**Background:**

Studies have indicated that cystic lesions play a crucial role in the repair processes of steroid-induced osteonecrosis of the femoral head and its subsequent collapse. Here, we employed single-cell RNA sequencing (scRNA-seq) technology to investigate the transcriptomic landscape and repair mechanisms of cystic lesions in SIONFH.

**Methods:**

We applied scRNA-seq combined with computational approaches to characterize distinct cell subsets and their molecular signatures within cystic lesions from three SIONFH patients. Additionally, histological assays were conducted to observe pathological manifestations of these lesions.

**Results:**

Eight cell types were identified in cystic lesions of SIONFH. Among them, chondrocytes were divided into five subgroups. Among them, chondrocytes were divided into five subgroups: homeostatic chondrocytes (HomC), fibrocartilage chondrocytes (FC), prehypertrophic chondrocytes (preHTC), inflammatory chondrocytes (InflamC), and hypertrophic chondrocytes (HTC). Additionally, histological assays showed the presence of chondrocytes and a transition zone from chondrocytes to bone tissue within the cystic lesions. Notably, we report that one of the HTC clusters with CLIC3+ expression exhibited a strong involved in bone mineralization, osteoblast differentiation, and cell differentiation.

**Conclusion:**

We have delineated the cellular heterogeneity and molecular signatures of cystic lesions in SIONFH. The results reveal a distinct repair program within these lesions, which might be driven by chondrocyte hypertrophy and might culminate in osteogenic differentiation.

## 1 Introduction

Steroid-induced osteonecrosis of the femoral head (SIONFH) represents a predominant subtype of non-traumatic ONFH, constituting nearly 40% of clinical cases (1, 2). This debilitating orthopedic condition arises primarily from prolonged high-dose glucocorticoid exposure - an unavoidable therapeutic necessity for managing various systemic diseases despite its iatrogenic consequences. The multifactorial pathogenesis involves complex interplay among genetic susceptibility, vascular compromise, metabolic disturbances (particularly lipid dysregulation), and elevated intraosseous pressure, collectively culminating in ischemic bone marrow necrosis and subsequent femoral head collapse (3). Characteristic radiographic progression manifests through three hallmark features: initial crescent sign formation, development of cystic lesions, and ultimately articular surface collapse - the latter serving as the definitive radiological threshold for advanced disease staging (4). This terminal collapse event precipitates rapid joint degeneration, leading to refractory hip pain, progressive mobility impairment, and in most cases, inevitable requirement for total hip arthroplasty.

Emerging evidence indicates that cystic lesions potentiate femoral head collapse risk, accelerate SIONFH progression, and adversely impact long-term prognostic outcomes (5). According to the Association Research Circulation Osseous (ARCO) staging system, cystic lesions represent a pathognomonic feature of Stage II osteonecrosis of the femoral head, radiographically characterized by subchondral radiolucent foci (6). Pathologically, these lesions predominantly consist of fibrous granulation tissue (7). The pathogenesis of cystic lesions formation remains elusive. Current models postulate that aberrant mechanical loading on sclerotic rims induces osteoclastic resorption, initiating a cystogenic cascade (8). It has been established that cystic lesions are associated with the collapse of the femoral head and disease prognosis in SIONFH (5). However, the reparative potential of cystic lesions in SIONFH remains largely unexplored. Lakhotia et al. observed varying degrees of new bone ingrowth in the cystic areas after femoral varus derotation osteotomy of ONFH patients using computed tomography (9). He et al. also found that spontaneous reparative transformation in subsets of cystic lesions during disease progression (10). These findings demonstrate that cystic lesions possess bone repair potential. Nevertheless, the underlying biological mechanisms remain to be elucidated.

Single-cell RNA sequencing (scRNA-seq) is a high-throughput sequencing technique that enables sequencing at the individual cell level (11). By sequencing the transcriptome of individual cells, scRNA-seq provides spatiotemporal dynamics of cellular processes, gene expression patterns, and cell heterogeneity. This technology possesses the capability to discern distinct cell types and acquire knowledge regarding physiological and pathological processes of certain disease. ScRNA-seq holds considerable clinical significance in domains such as oncology, immunology, and embryonic development (12–14). Presently, the majority of investigations on cystic lesions based on imaging and histological methods. Nevertheless, the utilization of scRNA-seq technology in studying cystic lesions remains unexplored.

Therefore, this study aims to systematically investigate the transcriptomic heterogeneity and aberrant repair mechanisms within cystic lesions of SIONFH using scRNA-seq technology. Our analysis delineates the cellular atlas of cystic lesions and identifies a pathological repair cascade characterized by chondrocyte hypertrophy with subsequent osteogenic differentiation, which might to be a hallmark of this unique microenvironment.

## 2 Materials and methods

### 2.1 Clinical specimen collection

All cystic lesions specimens were obtained from SIONFH patients undergoing total hip arthroplasty at the Third Affiliated Hospital of Guangzhou University of Chinese Medicine. Research protocols involving human subjects were approved by the Ethics Committee of the Third Affiliated Hospital of Guangzhou University of Chinese Medicine (approval number: GYH202101-04). All participants in this study were asked for Informed consent. The clinical and demographic characteristics of the patients were collected from medical records (**Supplementary Table 1**). The representative imaging data information are shown in **Supplementary Figure 1**.

### 2.2 Single-cell suspension preparation

After acquirement, fresh samples were washed with phosphate-buffered saline (PBS), then minced into approximately 1-millimeter pieces on ice. Tissue fragments were digested with 0.2% collagenase II (#2195526, Gibco, USA) in DMEM solution at 37°C for 2 h. The digested suspension was filtered through a 70-μm cell strainer. After centrifugation at 500 x g for 8 minutes, erythrocytes were lysed using erythrocyte lysis buffer.

### 2.3 Single-cell RNA-seq using 10x genomics chromium

According to the manufacturer’s instructions, the Chromium Single Cell 3e V2/V3 Kit was used to construct the scRNA-seq library. Briefly, cells were loaded into Chromium microfluidic chips with 3’ chemistry and barcoded using 10x Chromium Controller. Then, RNA from the barcoded cells underwent reverse transcription, followed by cDNA amplification and library construction. Sequencing was performed on an Illumina NovaSeq 6000 system.

### 2.4 Cell clustering and annotating

Gene expression levels were processed using the LogNormalize method of the”Normalization”function in the Seurat package. Dimensional reduction was conducted via principal components analysis (PCA). Batch effects were removed using Harmony (v0.1.0). The top 20 principal components were selected for clustering analysis in our default workflow. Identified clusters were visualized with the t-distributed stochastic neighbor embedding (t-SNE). Cell types were annotated by combining cellular biomarkers from previous articles and Gene Ontology (GO) analysis.

### 2.5 Gene enrichment analysis

Enrichment analysis included GO enrichment analysis and Kyoto Encyclopedia of Genes and Genomes (KEGG) enrichment analysis. Enrichment analysis were performed using the enrichGO and enrichKEGG functions of the clusterProfiler (V 3.18.0) R package.

### 2.6 Cell–cell communication analysis

To identify potential intercellular interactions, we performed cell–cell communication analysis using the CellPhoneDB Python package (version 2.1.7) (15). CellPhoneDB primarily utilizes the profiles information of receptors in one cell type and ligands in another cell type to infer interactions between different cell types. We identified the most relevant cell type-specific interactions between ligands and receptors and visualized the inferred intercellular communication networks for each ligand-receptor pair and each signaling pathway by circle plots.

### 2.7 Pseudotime analysis

Pseudotime analysis was performed using Monocle2 (version 2.14.0) to characterize chondrocyte differentiation (16). Per standard protocol, integrated gene expression data were imported into Monocle2, Cells were ordered based on differentially expressed genes (DEGs) between chondrocytes subclusters. Employing the DDRTree method for dimensionality reduction and using default parameters in Monocle2 for cell ordering, we inferred the differentiation trajectories of chondrocytes.

### 2.8 Histological analysis

Stereotactic whole section sampling of the femoral head was used. Each specimen were sectioned at 5-mm intervals, and the portion of each section containing cystic lesions was sampled. Specimens were fixed in 4% paraformaldehyde (PFA) solution (G1101–500 ML Servicebio, Wuhan, China) for 2 days at room temperature. After decalcified by 10% EDTA solution, samples were embedded in paraffin and serially cut into 5 µm-thick sections. Following a previously reported procedure, every fifth section of two sections were selected and stained with Safranin-O/fast green (#G1371, Solarbio, Beijing, China), hematoxylin and eosin (H&E) (#C0105, Beyotime), and Alisin blue (#C0155M, Beyotime) to evaluate histological characteristic (17). After that, the sections were observed under a digital section scanner (3DHISTEC, Pannoramic MIDI).

### 2.9 Immunofluorescence and immunohistochemical staining

The dewaxing and rehydration process was performed on paraffin-embedded sections.

To perform immunofluorescent staining, sections were blocked with endogenous catalase blocker for 15 min and 3% BSA (MP Biomedicals, Cat.# 0218054990) for 40 min. Then, sections were incubated overnight at 4°C with primary antibodies as follows: MMP13 (rabbit, 1:1000, 66034-1-IG, 2C8F3, PROTEINTECH GROUP, USA), COL10A1 (1:100; Proteintech Group, Inc), SPP1 (1:100; Proteintech Group, Inc) and RUNX2 (1:100; Proteintech Group, Inc). After that, sections were stained with secondary antibody for immunohistochemical staining (IHC) (horseradish peroxidase-conjugated anti-mouse&rabbit: absin, Cat.# 996, [1:200]) or immunofluorescence staining (IF) (Goat pABs to Rb IgG (Alexa Fluor^®^ 488): Abcam, Cat.# ab150081; Goat pABs to Ms IgG (Alexa Fluor^®^ 555): Abcam, Cat.# ab150118, [1:200]) for 2 h at room temperature. Sections were washed with PBST three times and stained with hematoxylin for IHC or DAPI (Invitrogen, Cat.# D1306) for IF. Sections were visualized with the confocal fluorescence microscope (LSM800, Carl Zeiss, Germany) or digital section scanner (3DHISTEC, Pannoramic MIDI).

### 2.10 Western blot

Femoral head bone samples from three femoral neck fracture (FNF) and three SIONFH patients were obtained after total hip arthroplasty. Cystic lesions and bone sample were collected and immediately frozen in liquid nitrogen. Tissue lysates were prepared using RIPA buffer (Beyotime, P0013B) with protease inhibitors(Beyotime, P0013B). Protein concentrations were determined by BCA assay (Beyotime, P0009). Samples (30 μg/lane) were resolved on 10% SDS-PAGE gels and transferred to PVDF membranes (Beyotime, FFP20). After blocking with 5% non-fat milk (Beyotime, P0216) in TBST (1 h, RT), membranes were incubated overnight at 4°C with primary antibodies: anti-SPP1 (1:1000; Abcam, ab58632), anti-RUNX2 (1:800; CST, #94808), anti-ColoA1 (1:800; CST, #94808), anti-MMP3(1:800; CST, #94808), anti-CLIC3 (1:800; CST, #94808), anti-GAPDH (1:5000; Proteintech, 60004-1-Ig). Following TBST washes (3 × 10 min), HRP-conjugated secondary antibodies (1:5000; Affinity, S0001) were applied (1 h, RT). Signals were detected with ECL substrate (Beyotime, P0018S) using a ChemiDoc MP System (Bio-Rad).

### 2.11 RT-qPCR

Total RNA isolation was conducted using TRIzol reagent (Invitrogen, Carlsbad, CA, USA) per manufacturer guidelines. Complementary DNA (cDNA) was synthesized employing a reverse transcription kit (Takara, Tokyo, Japan) according to established protocols. The polymerase chain reaction amplification profile comprised: initial denaturation (95°C, 1 min), followed by 40 cycles of denaturation (95°C, 20 s), primer annealing (55°C, 20 s), and extension (72°C, 30 s). GAPDH served as the endogenous control. All reactions were performed in triplicate, with relative quantification determined via the 2-ΔΔCq method. Primer sequences are documented in **Supplementary Table 2**.

### 2.12 Statistical analysis

SPSS software (version 23.0) was used to perform statistical analysis. Data were presented as mean values ± SD. Shapiro-Wilk test and Levene method were used for the estimation of the data normal distributions and homogeneity of variance, respectively. For the comparation of mean values between two groups, paired or unpaired two-tailed Student’s t test was used. One-way or two-way analysis of variance (ANOVA) followed by Tukey’s *post-hoc* tests were used to assess the statistical significance of the mean values of more than two groups. Differences with *p* < 0.05 indicates statistical significance.

## 3 Result

### 3.1 Cell clusters in cystic lesions

**Figure 1:**
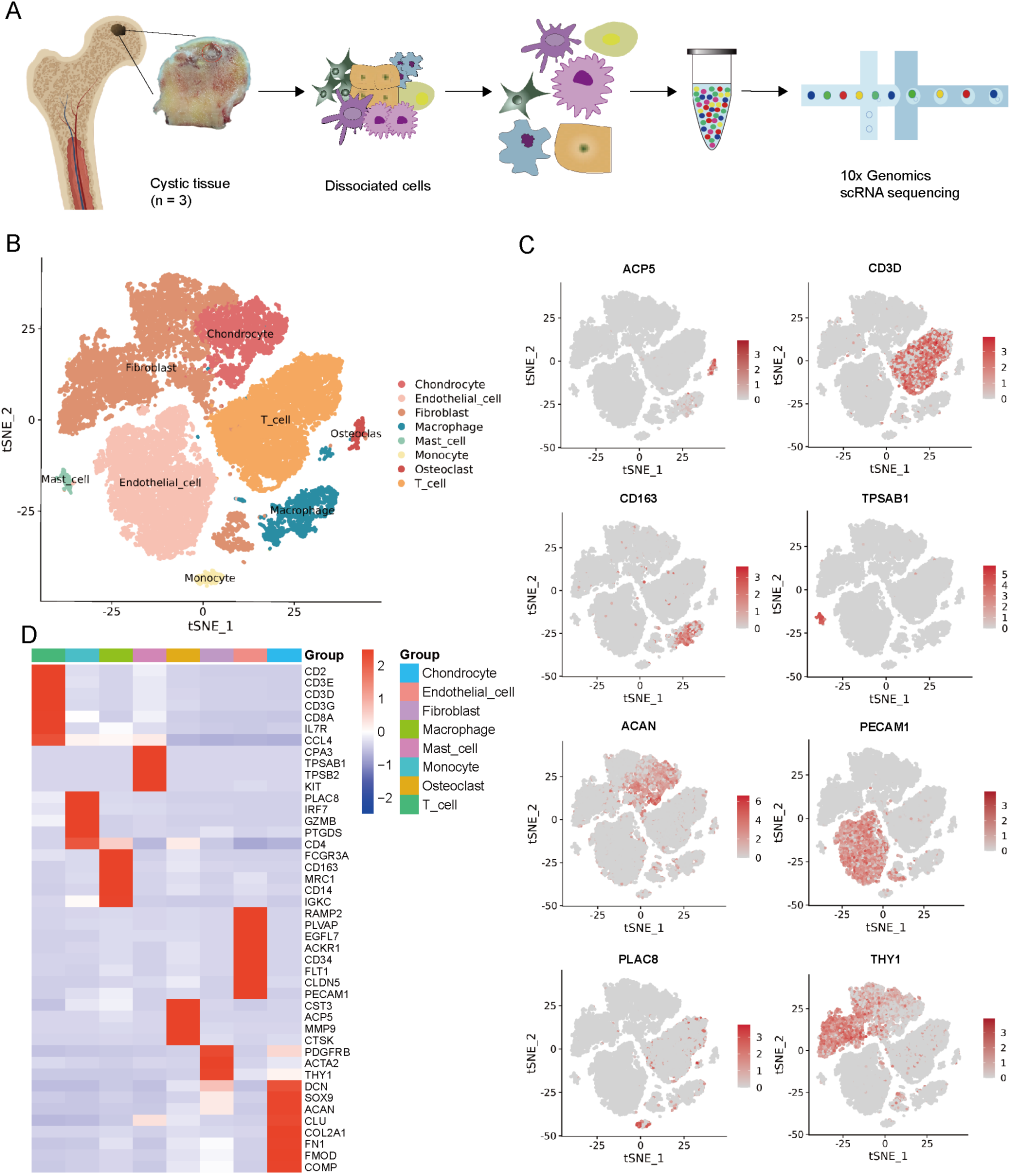
Single-cell RNA-seq of cystic lesions in SIONFH. **(A)** Schematic workflow of the experimental strategy. **(B)** t-SNE plot showing eight cell clusters in cystic lesions. **(C)** t-SNE plots showing eight cell type identified by marker gene expression (ACP5: osteoclasts; CD3D: T cells; CD163: macrophages; TPSAB1: mast cells; ACAN: chondrocytes; PECAM1: endothelial cells; PLAC8: monocytes cells; THY1: fibroblasts cells). **(D)** Heatmap plot showing scaled expression of canonical cell type-associated genes for clusters. SIONFH, steroid-induced osteonecrosis of the femoral head; scRNA-seq, Single-cell RNA sequencing; t-SNE, t-distributed stochastic neighbor embedding.

To determine cellular subtypes and their genetic characteristics, we obtained three cystic lesion specimens from the hip joints of three SIONFH patients and performed scRNA-seq analysis (**Figure 1A**). In total, 30,224 individual cells were obtained for subsequent analysis after strict quality filtration (**Supplementary Figure 2**). Based on t-SNE analysis, 16 distinct cluster cells were identified based on unbiased cell type recognition (**Supplementary Table 3**). Among them, eight major cell types were defined by specific marker genes, including endothelial cells (ECs) (expressing PECAM1, PLVAP, and FLT1), fibroblasts cells (FBs) (expressing THY1, PDGFRB, COL1A1, and COL1A2), chondrocytes (expressing ACAN, SOX9, and COL2A1), monocytes cells (expressing PLAC8), macrophages (expressing CD163, CD68, and MRC1), T cells (expressing CD3D, CD3E, and CD8A), mast cells (expressing TPSAB1, TPSB2, and KIT), and osteoclasts (expressing ACP5, CTSK, and MMP9) (**Figures 1B–D**). To gain a deeper understanding of the function on different cell types, GO enrichment analysis was conducted. Each cell type-specific gene displayed high enrichment in the GO terms expected (**Supplementary Figure 3**, **Supplementary Table 4**). For example, genes that are specifically expressed in ECs cluster are significantly enriched in angiogenesis and vasculature development; specific genes of chondrocyte cluster are significantly enriched in cartilage condensation and chondrocyte differentiation; specific genes of osteoclast cluster are significantly enriched in osteoclast differentiation. These analyses strongly support the accuracy of our cell‐type assignments.

### 3.2 Identification of FBs and ECs

**Figure 2:**
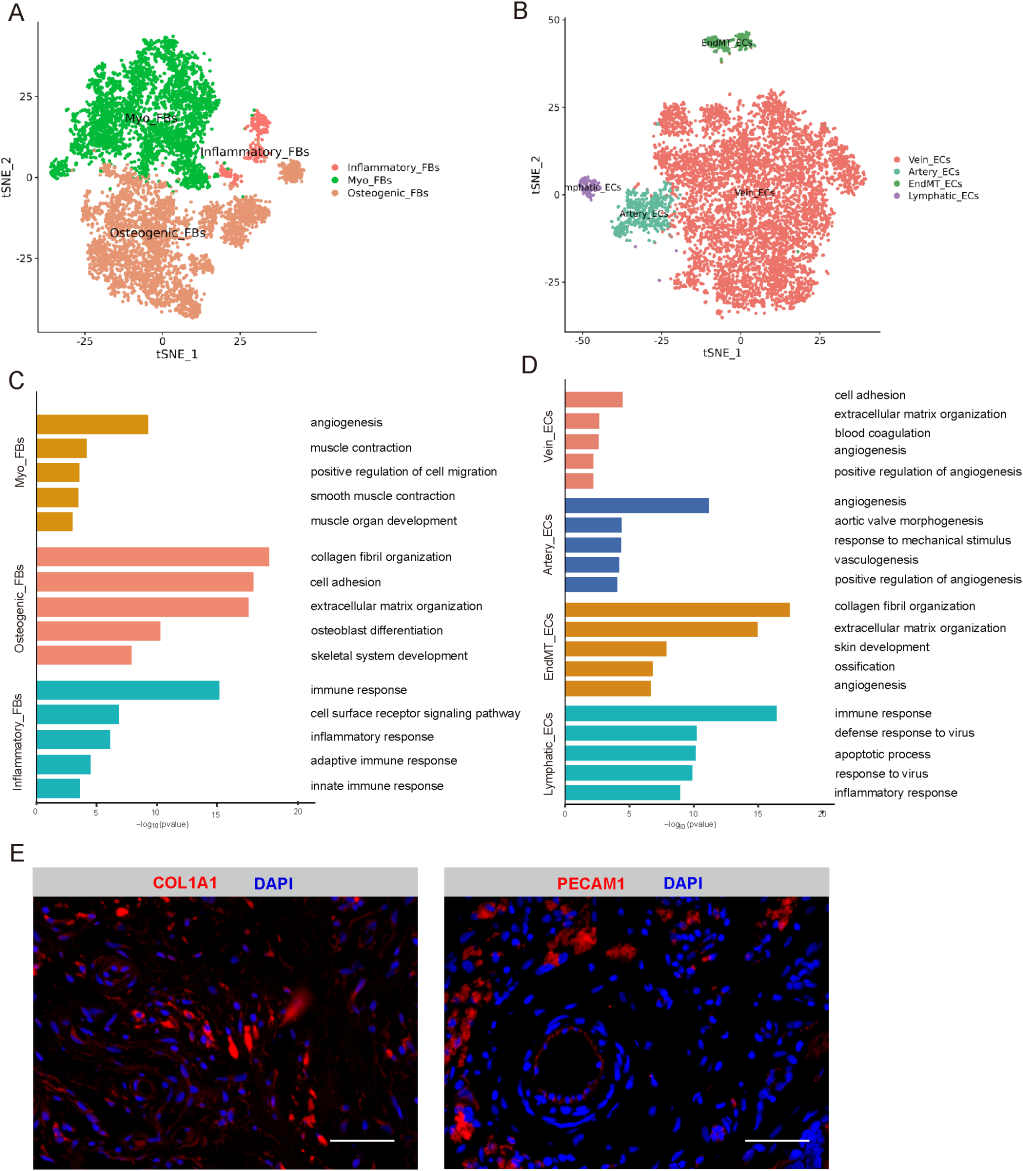
Identification of FBs and ECs. **(A)** t-SNE plot showing three subclusters of FBs. **(B)** t-SNE plot showing four subclusters of ECs. **(C)** The enriched GO terms results of marker genes of three cell subcluster of FBS. **(D)** The enriched GO terms results of marker genes of four cell subcluster of ECs. **(E)** Immunofluorescence expression of marker genes of FBs and ECs, respectively. Scale bar, 50 µm. t-SNE, t-distributed stochastic neighbor embedding; FBs, fibroblasts cells; ECs, endothelial cells; GO, gene ontology.

Through cluster analysis of the FBs, five subclusters (**Supplementary Figure 4A**) were identified. Among them, three major cell types were defined by specific marker genes. Myofibroblast cells (Myo_FBs) cluster was enriched in genes controlling the angiogenesis, muscle contraction, smooth muscle contraction, and muscle organ development, with high expression levels of ACTA2 and TAGLN (**Figures 2A, C**, **Supplementary Figures 4B, E**). Osteogenic fibroblast cells (Osteogenic_FBs) cluster was high in genes associated with collagen fibril organization, osteoblast differentiation, and skeletal system development, showing an upregulation of GJA1, MMP2, and COL1A1 (**Figures 2A, C**, **Supplementary Figures 4B, E**). Additionally, cells in the inflammatory fibroblast cells (Inflammatory_FBs) cluster were significantly enrich in immune response, cell surface receptor signaling pathway, and inflammatory response, with high expression levels of CXCR4, IL32, and PTPRC (**Figures 2A, C**, **Supplementary Figures 4B, E**).

To investigate ECs heterogeneity in cystic lesions, four subclusters (**Supplementary Figure 4C**) were identified. We identified four empirically defined populations: vein endothelial cells (Vein_ECs), artery endothelial cells (Artery_ECs), endothelial mesenchymal transition endothelial cells (EndMT_ECs), and lymphatic endothelial cells (Lymphatic_ECs) (**Figure 2B**). Vein_ECs was identified as cluster with high expression of genes related to blood coagulation and angiogenesis, including ACKR1 and VWF (**Figure 2D**, **Supplementary Figures 4D, F**). Artery_ECs were highly enriched in angiogenesis, aortic valve morphogenesis, and response to mechanical stimulus, with high expression with HEY1, IGFBP3, and EFNB2 (**Figure 2D**, **Supplementary Figures 4D, F**). EndMT_ECs cluster was enriched in genes associated with collagen fibril organization, extracellular matrix organization, and ossification, including COL1A1, COL1A2 and THY1 (**Figure 2D**, **Supplementary Figures 4D, F**). Additionally, cells in the Lymphatic_ECs cluster were mainly enriched in immune response, defense response to virus, and inflammatory response, with upregulated expression of CD69 and CXCR4 (**Figure 2D**, **Supplementary Figures 4D, F**). Immunofluorescence analysis confirmed the expression of these FB and EC markers in cystic lesions (**Figure 2E**).

### 3.3 Identification of macrophage cells

**Figure 3:**
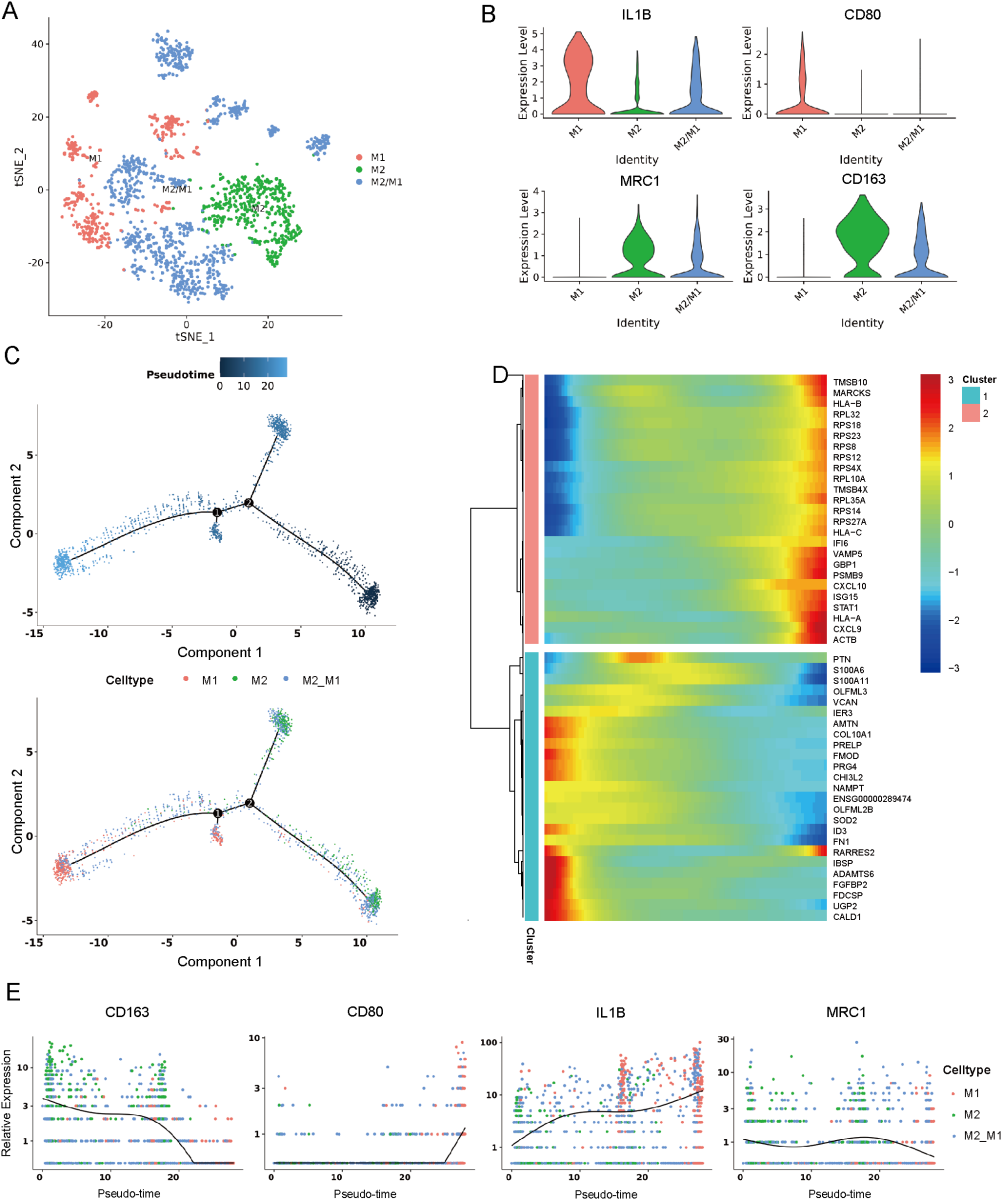
Identification of macrophage cells. **(A)** t-SNE plot showing three subclusters of macrophage cells. **(B)** Violin plots showing three cell type identified by marker gene expression (IL1B and CD80: M1 macrophages; MRC1 and CD163: M2 macrophages; IL1B, CD80, MRC1 and CD163: M2/M1). **(C)** Trajectory analysis of three subclusters of macrophage cells. **(D)** Dynamic pattern of gene expression changes along the entire lineage differentiation trajectory. **(E)** Expression of macrophage markers on a pseudo-time scale. t-SNE, t-distributed stochastic neighbor embedding.

Through cluster analysis of macrophages, 12 subclusters (**Supplementary Figure 5A**) were identified. Among them, three major cell types were defined by specific marker genes (**Figure 3A**), including M1 macrophages (expressing IL1B and CD80), M2 macrophages (expressing MRC1 and CD163), and M2/M1 macrophages (expressing IL1B, CD80, MRC1 and CD163) (**Figure 3B**, **Supplementary Figure 5B**) (18, 19). Trajectory analysis revealed that M2/M1 macrophages and M2 macrophages can transform into M1 macrophages over time (**Figure 3C**). A dynamic pattern of gene expression changes along the entire lineage differentiation trajectory is displayed in **Figure 3D**. The marker genes of M2/M1 macrophages and M2 macrophages such as MRC1 and CD163 decreased along pseudotime progression; while the marker genes of M1 macrophages such as CD80 and IL1B gradually increased along pseudotime progression (**Figure 3E**). Immunofluorescence analysis confirmed high CD163 expression in cystic lesions (**Supplementary Figure 5C**).

### 3.4 Identification of chondrocyte cells

**Figure 4:**
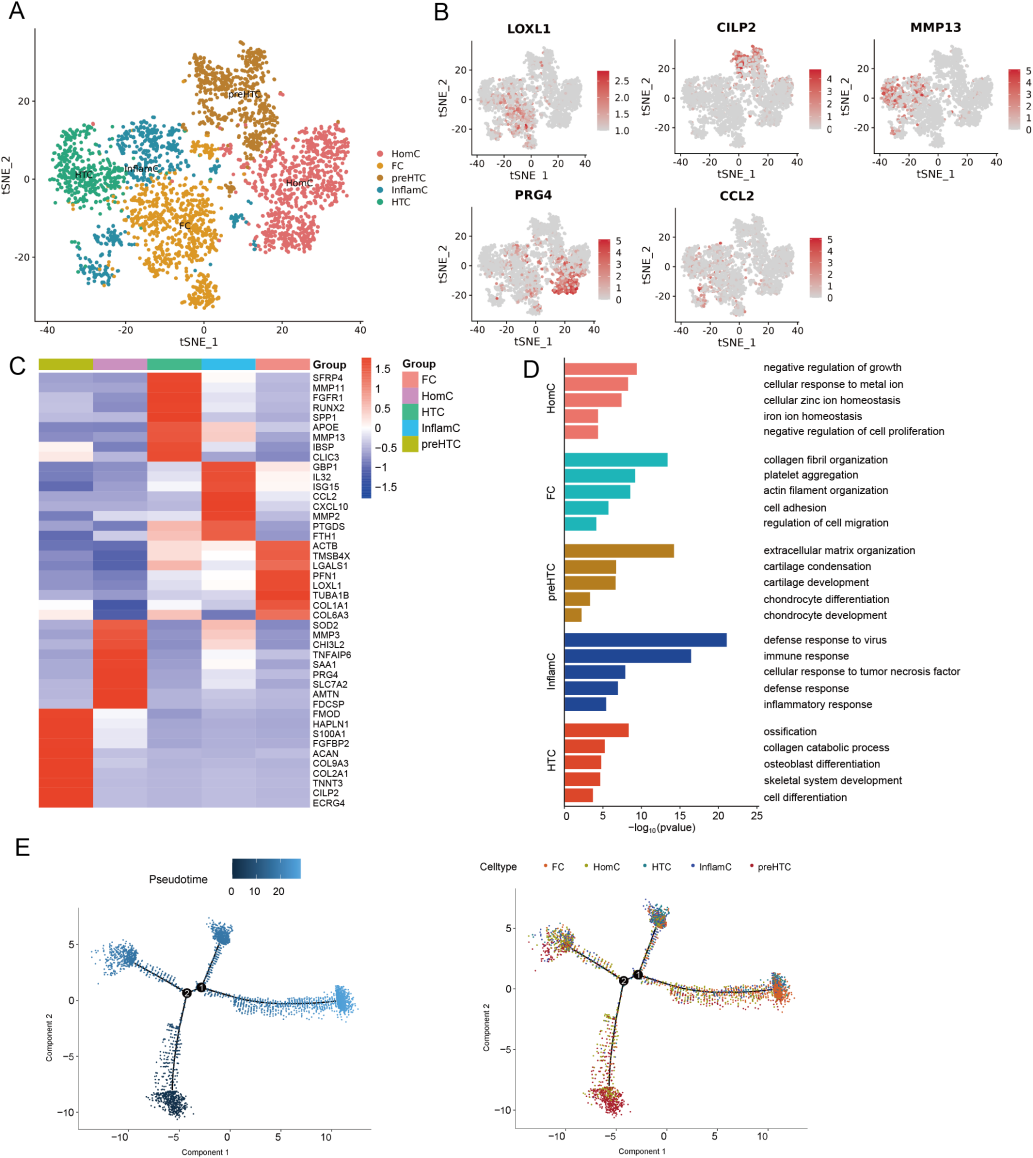
Identification of chonrocytes cells. **(A)** t-SNE plot showing five subclusters of chonrocytes cells. **(B)** t-SNE plots showing five cell type identified by marker gene expression (LOXL1: FC; CILP2: preHTC; MMP13: HTC; PRG4: HomC; CCL2: InflamC). **(C)** Heatmap plot showing scaled expression of canonical cell type-associated genes for five subclusters of chonrocytes cells. **(D)** The enriched GO terms results of marker genes of five subcluster of chonrocytes cells. **(E)** Trajectory analysis of five subcluster of chonrocytes cells. t-SNE, t-distributed stochastic neighbor embedding; FC, fibrocartilage chondrocytes; preHTC, prehypertrophic chondrocytes; HTC, hypertrophic chondrocytes; HomC, homeostatic chondrocytes; InflamC, inflammatory chondrocytes; GO, gene ontology.

**Figure 5:**
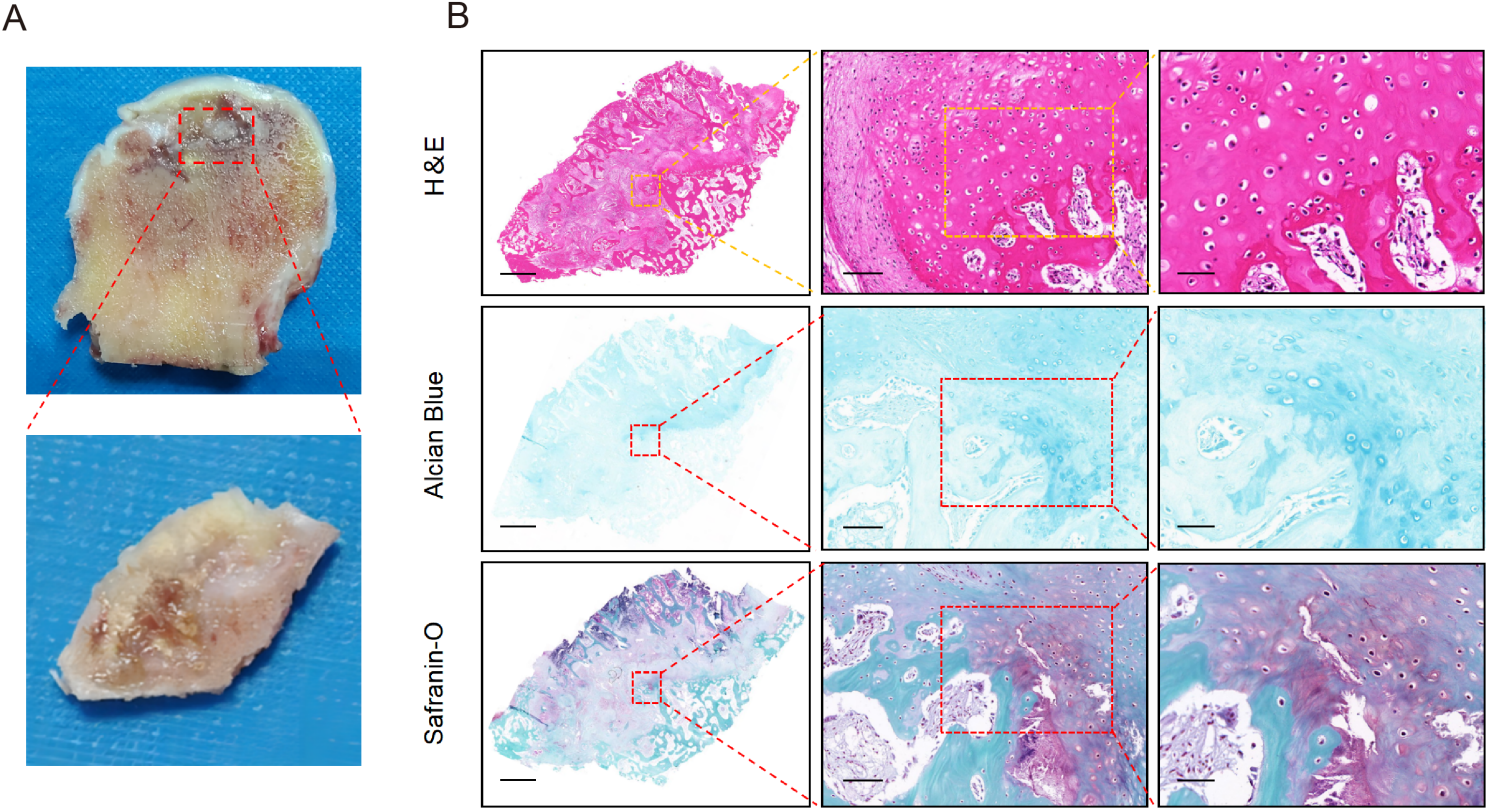
The pathological manifestations of cystic lesions. **(A)** The macroscopic appearance of cystic lesions in femoral head from SIONFH patients. **(B)** The results of H&E staining, Alcian blue staining, and Safranin O-Fast Green staining. Scale bar: left, 2.5 mm; middle, 100 µm; right, 50 µm. SIONFH, steroid-induced osteonecrosis of the femoral head.

To determine the identify of chondrocytes clusters, we identified five putative subclusters based on several transcriptomic studies of human chondrocytes, including: homeostatic chondrocytes (HomC), fibrocartilage chondrocytes (FC), prehypertrophic chondrocytes (preHTC), inflammatory chondrocytes (InflamC), and hypertrophic chondrocytes (HTC) (**Figure 4A**) (20, 21). Representative markers for HomC, FC, preHTC, InflamC, and HTC were revealed (**Figures 4B, C**), representing new combinations of genes to distinguish chondrocyte populations. In addition, the GEO term revealed that HomC cluster was mainly enriched in negative regulation of growth and cellular zinc ion homeostasis; FC cluster was enriched in collagen fibril organization and actin filament organization; preHTC cluster was highly enriched in cartilage condensation and chondrocyte differentiation; InflamC cluster with high expression of genes related to immune response and inflammatory response; HTC cluster was significantly enriched in ossification, collagen catabolic process, osteoblast differentiation, and cell differentiation (**Figure 4D**). To further validate the differentiation process of chondrocytes, pseudotime analysis was conducted. The pseudotime trajectory axis derived from Monocle indicated that preHTC were existed in the start of the trajectory and the front of HTC, InflamC and HomCs distributed along trajectory, HTC and FC were mainly existed in the end, suggesting that the pseudotime trajectory conforms to the regular pattern of chondrocytes differentiation (**Figure 4E**). Additionally, we performed H&E staining, Alcian blue staining, and Safranin O-fast green staining to evaluate histological characteristic of the cystic lesions (**Figure 5**). **Figure 5A** showed the cystic lesions obtained from necrosis femoral head. H&E staining showed the presence of a certain number of chondrocytes and HTC at the edge of the cystic lesions (**Figure 5B**). Alcian blue and Safranin O-fast green staining indicated that these cells were surrounded by a certain amount of cartilage matrix (**Figure 5B**). Meanwhile, we found a transition from cartilage tissue to bone matrix, suggesting the presence of a transitional zone from cartilage to bone tissue within the cystic lesions (**Figure 5B**).

### 3.5 Identification of HTC

**Figure 6:**
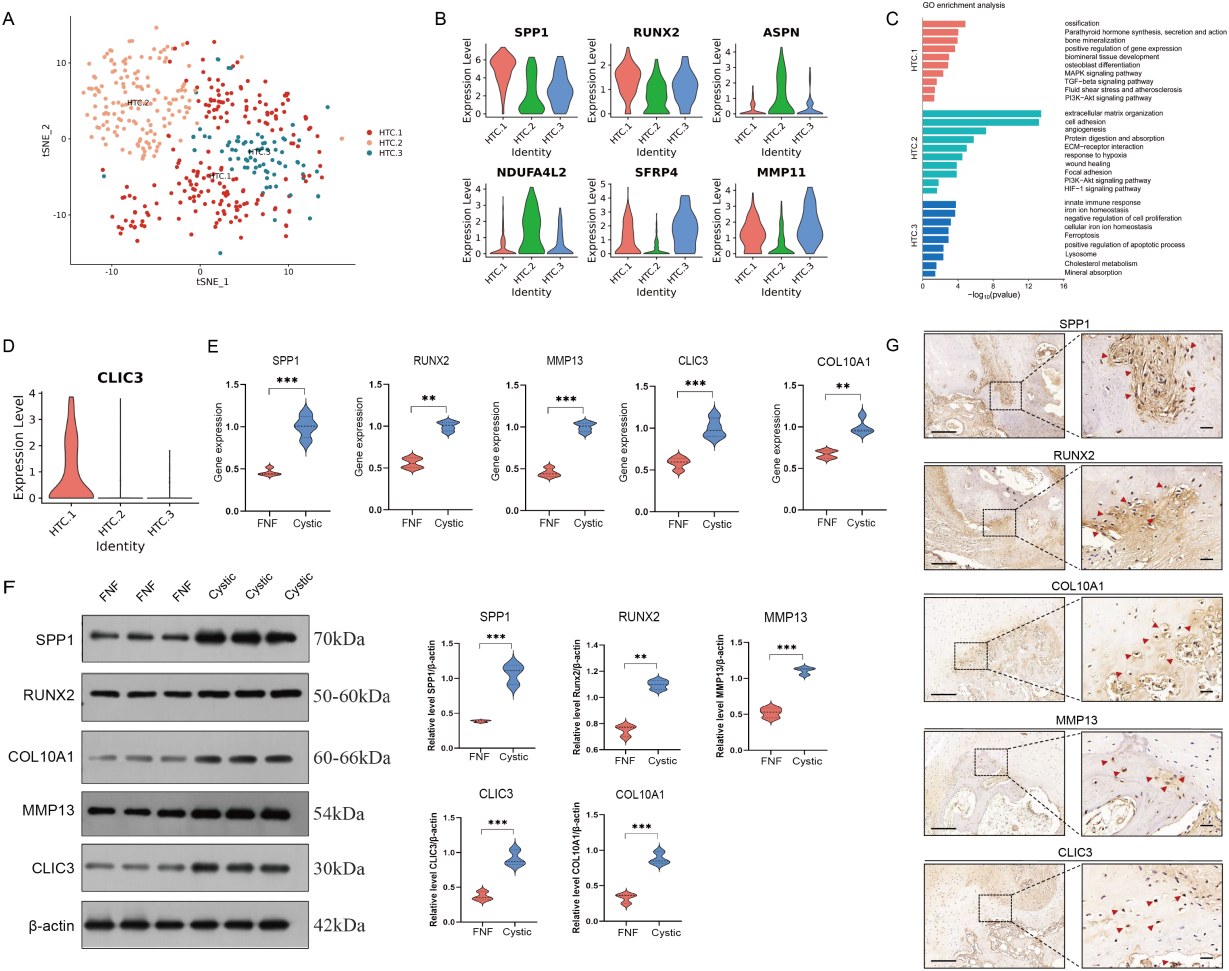
Identification of HTC cells. **(A)** t-SNE plot showing three subclusters of HTC. **(B)** Violin plots showing three cell type identified by marker gene expression (SPP1 and RUNX2: HTC.1; ASPN and NDUFA4L2: HTC.2; SFRP4 and MMP11: HTC.3). **(C)** The enriched GO terms results of marker genes of three subcluster of HTC. **(D)** The expression level of CLIC3 was significantly increased in HTC.1 cluster. **(E)** RT-qPCR analyses revealed that the gene expression levels of SPP1, RUNX2, COL10A1, MMP13, and CLIC3 were elevated in cystic lesions compared to those in FNF bone samples. **(F)** Western blot analyses revealed that the gene protein expression levels of SPP1, RUNX2, COL10A1, MMP13, and CLIC3 were elevated in cystic lesions compared to those in FNF bone samples. **(G)** Immunohistochemical expression of SPP1, RUNX2, COL10A1, MMP13, and CLIC3 in cystic lesions. Scale bar: left, 250 µm; right, 50 µm. HTC, hypertrophic chondrocytes; GO, gene ontology. **p < 0.01; ***p < 0.001.

To characterize the features of the HTC cluster, we divided the HTC cluster into three subclusters (**Figure 6A**). Among them, HTC.1 was identified as osteogenics-related cluster with high expression of genes enriched in ossification, bone mineralization, and osteoblast differentiation, MAPK and PI3K-Akt signaling pathway, with high expression levels of SPP1, RUNX2, and MMP13 (**Figures 6B, C**). HTC.2 cluster expressed unique markers that enriched in extracellular matrix organization and protein digestion and absorption, including ASPN and NDUFA4L2 (**Figures 6B, C**). HTC.3 cluster expressed high levels of SFRP4 and MMP11, with functional enrichment in immune response, iron ion homeostasis, and negative regulation of cell differentiation, along with KEGG pathway enrichment in ferroptosis signaling (**Figures 6B, C**). In order to explore the possible mechanisms of endochondral ossification for HTC, we further detected the significant key genes involved in this process. CLIC3, a marker gene associated with chloride ion channel, was calculated via DEGs by comparing HTC.1 to HTC.2 and HTC.3 (**Figure 6D**). There is a great difference in the expression multiples between three clusters of HTC, indicating that CLIC3 is a specific gene for regulating the differentiation of HTC into osteoblasts. Western blot and RT-qPCR analyses revealed that both protein and gene expression levels of osteogenic markers (SPP1 and RUNX2), hypertrophic chondrocyte (HTC) markers (COL10A1 and MMP13), and CLIC3 were markedly elevated in cystic lesions compared to those in bone samples from FNF patients (**Figures 6E, F**). Immunohistochemical analysis revealed a high expression of osteogenic markers (SPP1 and RUNX2), HTC markers (COL10A1 and MMP13), and CLIC3 at the edge of the cystic lesions (**Figure 6G**). The results of immunofluorescence analysis were consistent with that of immunohistochemical analysis (**Supplementary Figure 6**).

### 3.6 Cellular interaction in cystic lesions

**Figure 7:**
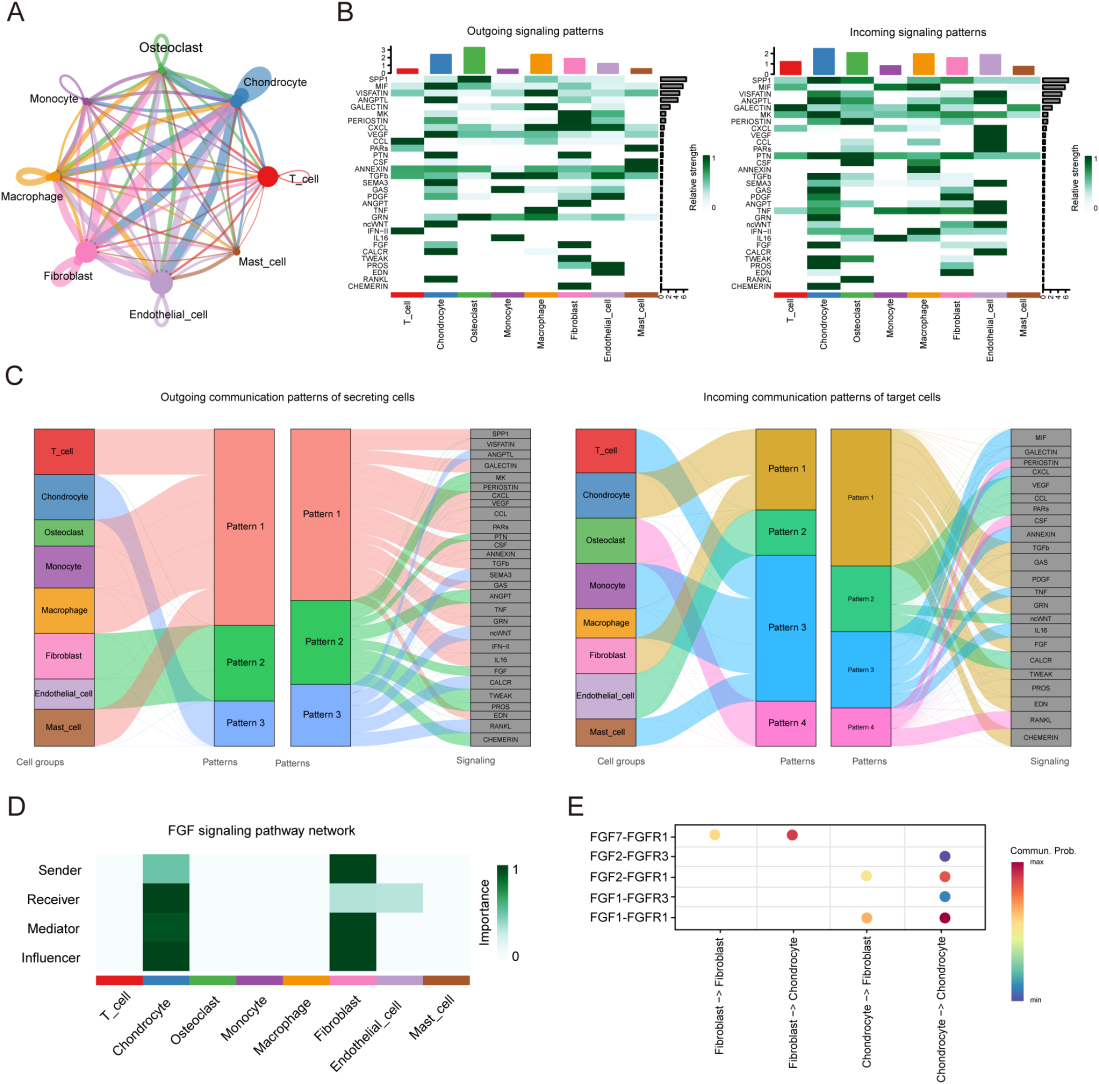
CellChat analysis of the communications between eight cell types in cystic lesions. **(A)** Analysis of the number of interactions among different cell types. **(B)** Overview of the outgoing signaling and incoming signaling of eight cell types. **(C)** Overview of the outgoing communication patterns of secreting cells and incoming communication patterns of target cells. **(D)** Communication roles played by eight cell types in FGF signaling pathway. **(E)** Bubble plot showing the ligand-receptor between fibroblasts and chondrocytes.

We identified eight types of cells, including four types of immune cells, repair-related cells like fibroblasts and endothelial cells and other cells like chondrocyte and osteoclast. Through CellChat analysis, we obtained a network map of interactions between cells (**Figure 7A**), and detected 75 significant ligand-receptor pairs among these 8 cell types, further categorizing them into 31 signaling pathways, including SPP1, MIF, VISFATIN, VEGF, CXCL, CCL, and MIF pathways (**Figure 7B**). The output of this analysis is a set of communication patterns linking cell populations with signaling pathways, whether in the context of output signals (treating cells as senders) or input signals (treating cells as receivers). The application of this analysis revealed three outgoing signaling patterns and four incoming signaling patterns (**Figure 7C**). For instance, the output indicated that all immune cells and osteoclasts exhibit outgoing signals characterized by pattern 1, representing multiple pathways including but not limited to SPP1, GALECTIN, CXCL, VEGF, and CCL. Fibroblasts and endothelial cells exhibit outgoing signals characterized by pattern 2, representing MK, PERIOSTIN, PTN, ANGPT, FGF among others. Chondrocytes exhibit outgoing signals characterized by pattern 3, representing ANGPTL, SEMA3, ncWNT among others. On the other hand, the communication signal patterns of target cells show that incoming signals from all immune cells are characterized by pattern 3, representing MIF, GALECTIN, CXCL, ANNEXIN, TNF, IL16 among others. Incoming signals from chondrocytes and fibroblasts are characterized by pattern 1, including TGFβ, GAS, PDGF, FGF among others. Incoming signals from endothelial cells are characterized by pattern 2. Incoming signals from osteoclasts are characterized by pattern 4, representing CSF, RANKL among others. We also found the FGF signaling network inferred by CellChat indicates that chondrocytes are the main source of receptors (**Figure 7D**). It exhibits weak autocrine activity in chondrocytes, primarily achieved through the ligand-receptor pair FGF1/FGFR1, but mainly secreted by fibroblasts, primarily through the ligand-receptor pair FGF7/FGFR1 (**Figure 7E**).

## 4 Discussion

**Figure 8:**
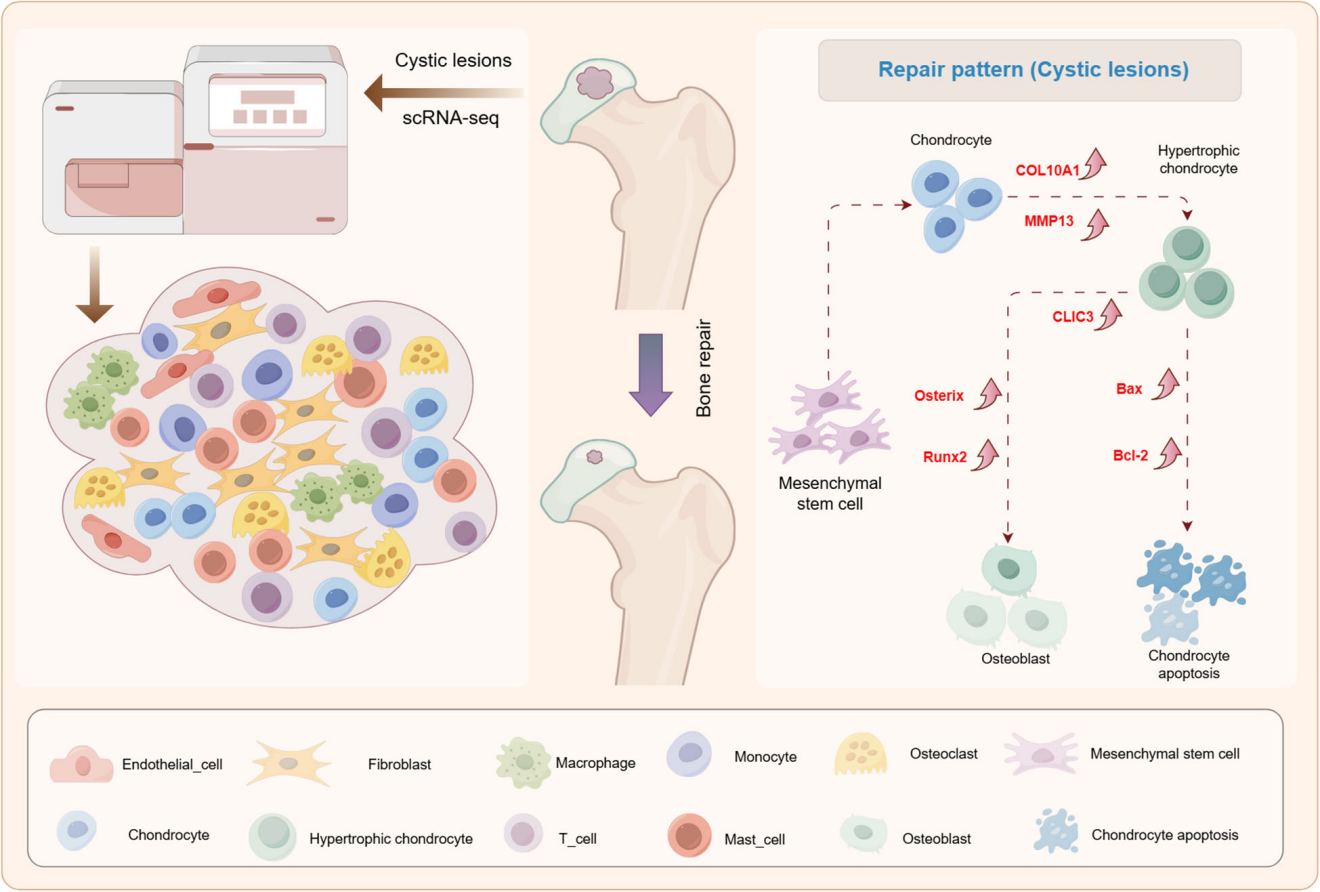
A graphical scheme of revealing the single-cell transcriptomic landscape of cystic lesions and the existence of a repair pattern involving chondrocyte hypertrophy and subsequent osteogenic differentiation in cystic lesions in SIONFH.

SIONFH is an intractable orthopedic condition with disability risk (22, 23). Current conventional approaches focusing on imaging feature identification and clinical symptom management in hip preservation therapy have demonstrated limited efficacy. Emerging evidence suggests that targeting necrotic femoral head repair mechanisms may represent a promising therapeutic strategy. Previous studies have demonstrated the regenerative capacity of cystic lesions, yet their underlying pathophysiology and repair mechanisms remain unknown (9, 10). In this study, we employed scRNA-seq technology to characterize the transcriptome profile of cystic lesions for the first time. Significantly, we identified a novel osteogenic differentiation repair pattern mediated by hypertrophic chondrocytes, providing mechanistic insights into femoral head regeneration (**Figure 8**).

Eight cell populations were identified in cystic lesions, including endothelial cells, fibroblasts, chondrocytes, tissue stem cells, macrophages, T cells, osteoclasts, and monocytes. Previous studies have indicated that the pathological characteristics of cystic lesions include fibrous granulation tissue (7). Consistent with this, gross examination of numerous necrotic femoral head specimens revealed substantial red or grayish-white tissue within the cystic lesion areas, exhibiting histological characteristics consistent with granulation or fibrotic tissue. Quantitative analysis confirmed fibroblasts as the predominant cellular component, validating the scRNA-Seq methodology employed in this study. Subgroup analysis of fibroblasts identified three distinct subtypes: Myo_FBs, Osteogenic_FBs, and Inflammatory_FBs. Several reports suggested that fibroblasts possess intrinsic osteogenic differentiation capacity independent of pluripotent stem cell transition (24, 25). Our scRNA-Seq data suggest active fibroblast participation in osseous repair processes within cystic lesions, though the exact molecular mechanisms require further elucidation. The results also revealed a significant proportion of endothelial cells. Studies have shown a close relationship between angiogenesis and bone formation, where H-type blood vessels have shown potential to induce bone formation, concurrent with higher expression levels of EMCN and PECAM-1 within these vessels (26). Therefore, promoting neovascularization represents a promising therapeutic strategy to prevent femoral head collapse in SIONFH. We found that both Vein_ECs and Artery_ECs are highly enriched in angiogenesis biological processes within the endothelial cell subgroups, which may contribute to the repair of cystic change in SIONFH.

Accumulating evidence in recent years highlights the indispensable role of bone immunology in the pathogenesis of SIONFH (27, 28). Immune cells can regulate the activation and function of various bone cells (osteoblasts, osteoclasts, mesenchymal stem cells, etc.), thereby regulating the processes of bone formation and repair (29). In this study, we observed significant immune cell infiltration in cystic lesions, including T cells, macrophages, monocytes, and mast cells. Consistent with previous studies, histological and bioinformatics analyses of ONFH revealed that the necrotic tissue contains a large number of immune cell infiltrations, such as macrophages, T lymphocytes, and B lymphocytes, implicating immune cells in ONFH onset and progression (30–32). The stimulation and inhibition of osteoblasts and osteoclasts by T cells are closely related to T cell subpopulations and cytokines, mainly achieved through the RANKL/RANK/OPG system (27). Macrophages, a type of “proliferative” immune cell, play a key role in the innate immune response. Macrophages can be “polarized” into different phenotypes based on their microenvironment, that is, “classically activated” M1 macrophages have pro-inflammatory effects, while “selectively activated” M2 macrophages have anti-inflammatory effects (33). Building on this, we identified three macrophage subtypes within the lesions. Pseudotemporal analysis found that M2/M1 macrophages and M2 macrophages can be transformed into M1 macrophages over time, indicating that in the local immune microenvironment of SIONFH cystic lesions, macrophages continuously polarize towards M1, leading to an increase in the M1/M2 ratio and causing imbalance of bone immunity, which may be one of the pathogenic mechanisms of SIONFH. Curcumin can prevent inflammation-mediated osteocyte apoptosis in a mouse model of glucocorticoid-related femoral head necrosis by inhibiting the polarization of M1 macrophages (34). Mongolicin can regulate inflammation and promote bone formation by inhibiting the TLR4/NF-κB pathway and M1 macrophage polarization, reducing the incidence of femoral head osteonecrosis and alleviating pathological manifestations within the femoral head (35). Therefore, inhibiting M1 macrophage polarization represents a promising therapeutic strategy for SIONFH.

Interestingly, we identified chondrocytes within the cystic lesions. Our analysis distinguished five chondrocyte subtypes based on established markers from prior articular cartilage scRNA-seq studies (20, 21). The pseudotime trajectory axis derived from Monocle matched well with cell types and the cell arrangement in the pseudospace trajectory corresponded to spatial relationships of the cells. Bone formation occurs via intramembranous ossification or endochondral ossification (36). In endochondral ossification, there are three major models of transdifferentiation of chondrocytes to osteoblasts. Recently, growing evidences revealed that chondrocytes will mature and hypertrophy, and that they do not apoptose, but instead differentiate directly into osteoblasts and subsequently into osteocytes (37, 38). Consistent with this, we found that HTC were enriched in ossification, collagen catabolic process, and osteoblast differentiation. Cluster analysis revealed a distinct HTC.1 subpopulation with significant involvement in ossification and bone mineralization. We further conducted pathological observations on the cystic lesions. Through H&E, Alcian blue, and Safranin O-fast green staining, we observed the presence of chondrocytes and HTC and a transition zone from chondrocytes to bone tissue within the cystic lesions. The results of immunohistochemistry and immunofluorescence also indicated high expression of HTC markers and osteogenesis-related gene at the edge of the cystic lesions. Numerous studies have revealed that a multitude of signaling and transcription factors are critical for endochondral bone formation, including BMP (Bone Morphogenetic Proteins) signaling, Wnt/β-catenin signaling, Indian hedgehog (Ihh) pathway, and FGF signaling (39–41). Lyu et al. demonstrated that intraperitoneal injection of the BMP signaling inhibitor LDN193189HOU in mice rescues osteogenic activation in chondrocytes (42). Runx2, a core regulatory gene of osteogenic differentiation, exhibits dual functionality during chondrocyte-to-osteoblast transition: promoting chondrocyte hypertrophy while simultaneously activating osteoblast differentiation programs. Previous studies confirm that excessive activation of Runx2 accelerates chondrocyte catabolism (43). Consistent with this, our findings via Western blot, RT-qPCR, and immunohistochemistry analyses showed significantly elevated Runx2 expression levels within cystic lesions regions. Meanwhile, expression of the hypertrophic marker COL10A1 and catabolic enzyme MMP13 was markedly increased, providing direct evidence for chondrocyte hypertrophy and osteogenic transdifferentiation within cystic lesions. Notably, we found that a type of CLIC3^+^HTC might be associated with ossification, indicating that these cells are potentially involved in osteogenic process in cystic lesions. CLIC3 is a soluble protein that regulates chloride conductance and cell growth. Previous study reported that CLIC3 is a key gene modulating osteoblast differentiation and enhancing bone formation (44). It may regulate osteoblast differentiation via cytoskeleton-associated signaling processes. In addition, the KEGG analysis showed that HTC.1 were enriched in MAPK signaling pathway, indicating that CLIC3 may regulate osteoblast differentiation through MAPK signaling pathway, but this requires further investigation.

The strength of this study is that we provide the first single-cell transcriptomic landscape of cystic lesions in SIONFH. In addition, the results of scRNA-seq analysis revealed a repair pattern of HTC differentiating into osteoblast in cystic lesions. In terms of limitations, first, the sample size is relatively small, and subsequent scRNA-seq studies will require more samples. Second limitation is the lack of an animal model for cystic changes, which prevents us from validating our findings *in vivo*. Third, although our findings delineate a repair pattern in SIONFH cystic lesions, the current cohort was confined to Chinese patients; future multi-ethnic studies are required to verify this pattern’s universality.

## 5 Conclusion

In summary, we have delineated the cellular heterogeneity and molecular signatures of cystic lesions in SIONFH. Our research reveal a distinct repair program within these lesions, which might be driven by chondrocyte hypertrophy and might culminate in osteogenic differentiation. These findings will provide novel therapeutic targets for the repair of necrotic bone in SIONFH.

## Data Availability

The data presented in this study have been deposited in the Gene Expression Omnibus (GEO) repository under accession number GSE290411. These data are publicly available.

## References

[1] Birla V Vaish A Vaishya R. Risk factors and pathogenesis of steroid-induced osteonecrosis of femoral head - A scoping review. J Clin Orthop Trauma. (2021) 23:101643. doi: 10.1016/j.jcot.2021.101643, PMID: 34722150 PMC8531658

[2] Zhang J Cao J Liu Y Zhao L. Advances in the pathogenesis of steroid-associated osteonecrosis of the femoral head. Biomolecules. (2024) 14:667. doi: 10.3390/biom14060667, PMID: 38927070 PMC11202272

[3] Motta F Timilsina S Gershwin ME Carlo S. Steroid-induced osteonecrosis. J Transl Autoimmun. (2022) 5:100168. doi: 10.1016/j.jtauto.2022.100168, PMID: 36213422 PMC9535426

[4] Shi S Luo P Sun L Xie L Yu T Wang ZC. Prediction of the progression of femoral head collapse in ARCO stage 2-3A osteonecrosis based on the initial bone resorption lesion. Br J Radiol. (2021) 94:20200981. doi: 10.1259/bjr.20200981, PMID: 33125270 PMC7774699

[5] Zhang Z Lin T Zhong Y Song W Yang P Wang D. Effect of femoral head necrosis cystic area on femoral head collapse and stress distribution in femoral head: A clinical and finite element study [published correction appears in Open Med (Wars). Open Med (Wars). (2022) 17:1282–91. doi: 10.1515/med-2022-0506, PMID: 35892078 PMC9281584

[6] Sugano N Atsumi T Ohzono K Kubo T Hotokebuchi T Takaoka K. The 2001 revised criteria for diagnosis, classification, and staging of idiopathic osteonecrosis of the femoral head. J Orthop Sci. (2002) 7:601–5. doi: 10.1007/s007760200108, PMID: 12355139

[7] Resnick D Niwayama G Coutts RD. Subchondral cysts (geodes) in arthritic disorders: pathologic and radiographic appearance of the hip joint. AJR Am J Roentgenol. (1977) 128:799–806. doi: 10.2214/ajr.128.5.799, PMID: 404905

[8] Gao F Han J He Z Li Z. Radiological analysis of cystic lesion in osteonecrosis of the femoral head. Int Orthop. (2018) 42:1615–21. doi: 10.1007/s00264-018-3958-z, PMID: 29704023

[9] Lakhotia D Swaminathan S Shon WY Moon JG Dwivedi C Hong SJ. Healing process of osteonecrotic lesions of the femoral head following transtrochanteric rotational osteotomy: A computed tomography-based study. Clin Orthop Surg. (2017) 9:29–36. doi: 10.4055/cios.2017.9.1.29, PMID: 28261424 PMC5334024

[10] He XM He MC Yang P Zhang QW Chen ZQ He W. The therapeutic effect of huo xue tong luo capsules in association research circulation osseous (ARCO) stage II osteonecrosis of the femoral head: A clinical study with an average follow-up period of 7.95 years. Front Pharmacol. (2021) 12:773758. doi: 10.3389/fphar.2021.773758, PMID: 34899331 PMC8652332

[11] Slovin S Carissimo A Panariello F Grimaldi A Bouche V Gambardella G. Single-cell RNA sequencing analysis: A step-by-step overview. Methods Mol Biol. (2021) 2284:343–65. doi: 10.1007/978-1-0716-1307-8_19, PMID: 33835452

[12] Tao Y Ma Y Gu L Zhang Y Zhang Q Zhou L. Single-cell RNA sequencing reveals Shen-Bai-Jie-Du decoction retards colorectal tumorigenesis by regulating the TMEM131-TNF signaling pathway-mediated differentiation of immunosuppressive dendritic cells. Acta Pharm Sin B. (2025) 15:3545–60. doi: 10.1016/j.apsb.2025.05.013, PMID: 40698132 PMC12278398

[13] Lin J Huang L Li W Xiao H Pan M. Unraveling the oxidative stress landscape in diabetic foot ulcers: insights from bulk RNA and single-cell RNA sequencing data. Biol Direct. (2025) 20:79. doi: 10.1186/s13062-025-00672-5, PMID: 40616164 PMC12232072

[14] Riegel D Romero-Fernández E Simon M Adenugba A Singer K Mayr R. Integrated single-cell profiling dissects cell-state-specific enhancer landscapes of human tumor-infiltrating CD8+ T cells. Mol Cell. (2023) 83:622–636.e10. doi: 10.1016/j.molcel.2022.12.029, PMID: 36657444

[15] Lv Z Han J Li J Guo H Fei Y Sun Z. Single cell RNA-seq analysis identifies ferroptotic chondrocyte cluster and reveals TRPV1 as an anti-ferroptotic target in osteoarthritis. EBioMedicine. (2022) 84:104258. doi: 10.1016/j.ebiom.2022.104258, PMID: 36137413 PMC9494174

[16] Qiu X Hill A Packer J Lin D Ma Y Trapnell C. Single-cell mRNA quantification and differential analysis with Census. Nat Methods. (2017) 14:309–15. doi: 10.1038/nmeth.4150, PMID: 28114287 PMC5330805

[17] Lihua C Zhiyin T. Microplastics aggravates rheumatoid arthritis by affecting the proliferation/migration/inflammation of fibroblast-like synovial cells by regulating mitochondrial homeostasis. Int Immunopharmacol. (2023) 120:110268. doi: 10.1016/j.intimp.2023.110268, PMID: 37201404

[18] Murray PJ. Macrophage polarization. Annu Rev Physiol. (2017) 79:541–66. doi: 10.1146/annurev-physiol-022516-034339, PMID: 27813830

[19] Tian Z Yang S. Integrating the characteristic genes of macrophage pseudotime analysis in single-cell RNA-seq to construct a prediction model of atherosclerosis. Aging (Albany NY). (2023) 15:6361–79. doi: 10.18632/aging.204856, PMID: 37421595 PMC10373969

[20] Ji Q Zheng Y Zhang G Hu Y Fan X Hou Y. Single-cell RNA-seq analysis reveals the progression of human osteoarthritis. Ann Rheum Dis. (2019) 78:100–10. doi: 10.1136/annrheumdis-2017-212863, PMID: 30026257 PMC6317448

[21] Li H Jiang X Xiao Y Zhang Y Zhang W Doherty M. Combining single-cell RNA sequencing and population-based studies reveals hand osteoarthritis-associated chondrocyte subpopulations and pathways. Bone Res. (2023) 11:58. doi: 10.1038/s41413-023-00292-7, PMID: 37914703 PMC10620170

[22] Wu CT Kuo FC Yen SH Lin PC Wang JW Lee M. Impaction bone grafting augmented with a wire coil by the lightbulb technique for osteonecrosis of the femoral head. J Arthroplasty. (2022) 37:2063–70. doi: 10.1016/j.arth.2022.04.034, PMID: 35490978

[23] Wei QS Hong GJ Yuan YJ Chen ZQ Zhang QW He W. Huo Xue Tong Luo capsule, a vasoactive herbal formula prevents progression of asymptomatic osteonecrosis of femoral head: A prospective study. J Orthop Translat. (2018) 18:65–73. doi: 10.1016/j.jot.2018.11.002, PMID: 31508309 PMC6718872

[24] Knaup I Symmank J Bastian A Neuss S Pufe T Jacobs C. Impact of FGF1 on human periodontal ligament fibroblast growth, osteogenic differentiation and inflammatory reaction *in vitro*. Einfluss von FGF1 auf Wachstum, osteogene Differenzierung und inflammatorische Stressreaktion humaner Parodontalligamentfibroblasten in *vitro* . J Orofac Orthop. (2022) 83:42–55. doi: 10.1007/s00056-021-00363-6, PMID: 34874457

[25] Ceylan M Schoenmaker T Hogervorst JMA Jansen IDC Schimmel IM Prins CM. Osteogenic differentiation of human gingival fibroblasts inhibits osteoclast formation. Cells. (2024) 13:1090. doi: 10.3390/cells13131090, PMID: 38994943 PMC11240541

[26] Kusumbe AP Ramasamy SK Adams RH. Coupling of angiogenesis and osteogenesis by a specific vessel subtype in bone. Nature. (2014) 507:323–8. doi: 10.1038/nature13145, PMID: 24646994 PMC4943525

[27] Ma J Ge J Gao F Wang B Yue D Sun W. The role of immune regulatory cells in nontraumatic osteonecrosis of the femoral head: A retrospective clinical study. BioMed Res Int. (2019) 2019:1302015. doi: 10.1155/2019/1302015, PMID: 31828086 PMC6886356

[28] Zhang Q Sun W Li T Liu F. Polarization behavior of bone macrophage as well as associated osteoimmunity in glucocorticoid-induced osteonecrosis of the femoral head. J Inflammation Res. (2023) 16:879–94. doi: 10.2147/JIR.S401968, PMID: 36891172 PMC9986469

[29] Saxena Y Routh S Mukhopadhaya A. Immunoporosis: role of innate immune cells in osteoporosis. Front Immunol. (2021) 12:687037. doi: 10.3389/fimmu.2021.687037, PMID: 34421899 PMC8374941

[30] Vicaş RM Bodog FD Fugaru FO Grosu F Badea O Lazar L. Histopathological and immunohistochemical aspects of bone tissue in aseptic necrosis of the femoral head. Rom J Morphol Embryol. (2020) 61:1249–58., PMID: 34171073 10.47162/RJME.61.4.26 PMC8343594

[31] Zhao J Zhang X Guan J Su Y Jiang J. Identification of key biomarkers in steroid-induced osteonecrosis of the femoral head and their correlation with immune infiltration by bioinformatics analysis. BMC Musculoskelet Disord. (2022) 23:67. doi: 10.1186/s12891-022-04994-7, PMID: 35042504 PMC8767711

[32] Wang B Gong S Shao W Han L Li Z Zhang Z. Comprehensive analysis of pivotal biomarkers, immune cell infiltration and therapeutic drugs for steroid-induced osteonecrosis of the femoral head. Bioengineered. (2021) 12:5971–84. doi: 10.1080/21655979.2021.1972081, PMID: 34488536 PMC8815624

[33] Viola A Munari F Sánchez-Rodríguez R Scolaro T Castegna A. The metabolic signature of macrophage responses. Front Immunol. (2019) 10:1462. doi: 10.3389/fimmu.2019.01462, PMID: 31333642 PMC6618143

[34] Jin S Meng C He Y Wang X Zhang Q Wang Z. Curcumin prevents osteocyte apoptosis by inhibiting M1-type macrophage polarization in mice model of glucocorticoid-associated osteonecrosis of the femoral head. J Orthop Res. (2020) 38:2020–30. doi: 10.1002/jor.24619, PMID: 32009245 PMC7496963

[35] Zhu D Yu H Liu P Yang Q Chen Y Luo P. Calycosin modulates inflammation via suppressing TLR4/NF-κB pathway and promotes bone formation to ameliorate glucocorticoid-induced osteonecrosis of the femoral head in rat. Phytother Res. (2021) 35:2824–35. doi: 10.1002/ptr.v35.5, PMID: 33484002

[36] Aghajanian P Mohan S. The art of building bone: emerging role of chondrocyte-to-osteoblast transdifferentiation in endochondral ossification. Bone Res. (2018) 6:19. doi: 10.1038/s41413-018-0021-z, PMID: 29928541 PMC6002476

[37] Xiong J Ma R Xie K Shan C Chen H Wang Y. Recapitulation of endochondral ossification by hPSC-derived SOX9+ sclerotomal progenitors. Nat Commun. (2025) 16:2781. doi: 10.1038/s41467-025-58122-9, PMID: 40118845 PMC11928506

[38] Shu HS Liu YL Tang XT Zhang XS Zhou B Zou W. Tracing the skeletal progenitor transition during postnatal bone formation. Cell Stem Cell. (2021) 28:2122–2136.e3. doi: 10.1016/j.stem.2021.08.010, PMID: 34499868

[39] Wang K Ma C Feng JQ Jing Y. The emerging role of cell transdifferentiation in skeletal development and diseases. Int J Mol Sci. (2022) 23:5974. doi: 10.3390/ijms23115974, PMID: 35682655 PMC9180549

[40] Houben A Kostanova-Poliakova D Weissenböck M Graf J Teufel S Mark K. β-catenin activity in late hypertrophic chondrocytes locally orchestrates osteoblastogenesis and osteoclastogenesis. Development. (2016) 143:3826–38. doi: 10.1242/dev.137489, PMID: 27621061 PMC5087647

[41] Salazar VS Gamer LW Rosen V. BMP signalling in skeletal development, disease and repair. Nat Rev Endocrinol. (2016) 12:203–21. doi: 10.1038/nrendo.2016.12, PMID: 26893264

[42] Lyu Z Da Y Liu H Wang Z Zhu Y Tian J. Chsy1 deficiency reduces extracellular matrix productions and aggravates cartilage injury in osteoarthritis. Gene. (2022) 827:146466. doi: 10.1016/j.gene.2022.146466, PMID: 35390446

[43] Wu X Lai Y Chen S Zhou C Tao C Fu X. Kindlin-2 preserves integrity of the articular cartilage to protect against osteoarthritis. Nat Aging. (2022) 2:332–47. doi: 10.1038/s43587-021-00165-w, PMID: 37117739

[44] Brum AM van der Leije CS Schreuders-Koedam M Verhoeven J Janssen M Dekkers DH. Identification of chloride intracellular channel protein 3 as a novel gene affecting human bone formation. JBMR Plus. (2017) 1:16–26. doi: 10.1002/jbm4.10003, PMID: 30283877 PMC6124162

