# Supplementary material for: Single-cell RNA-seq reveals a repair pattern in cystic lesions in steroid induced osteonecrosis of the femoral head
Source: Front Immunol. 2025 Sep 1;16:1626337. doi: 10.3389/fimmu.2025.1626337 (PMC12433953; doi:10.3389/fimmu.2025.1626337)
Supplement: Supplementary file 1 [file DataSheet1.docx]

**Supplementary information**

**Single-cell RNA-Seq reveals a repair pattern in cystic lesions in steroid induced osteonecrosis of the femoral head**

**Fig. S1.**


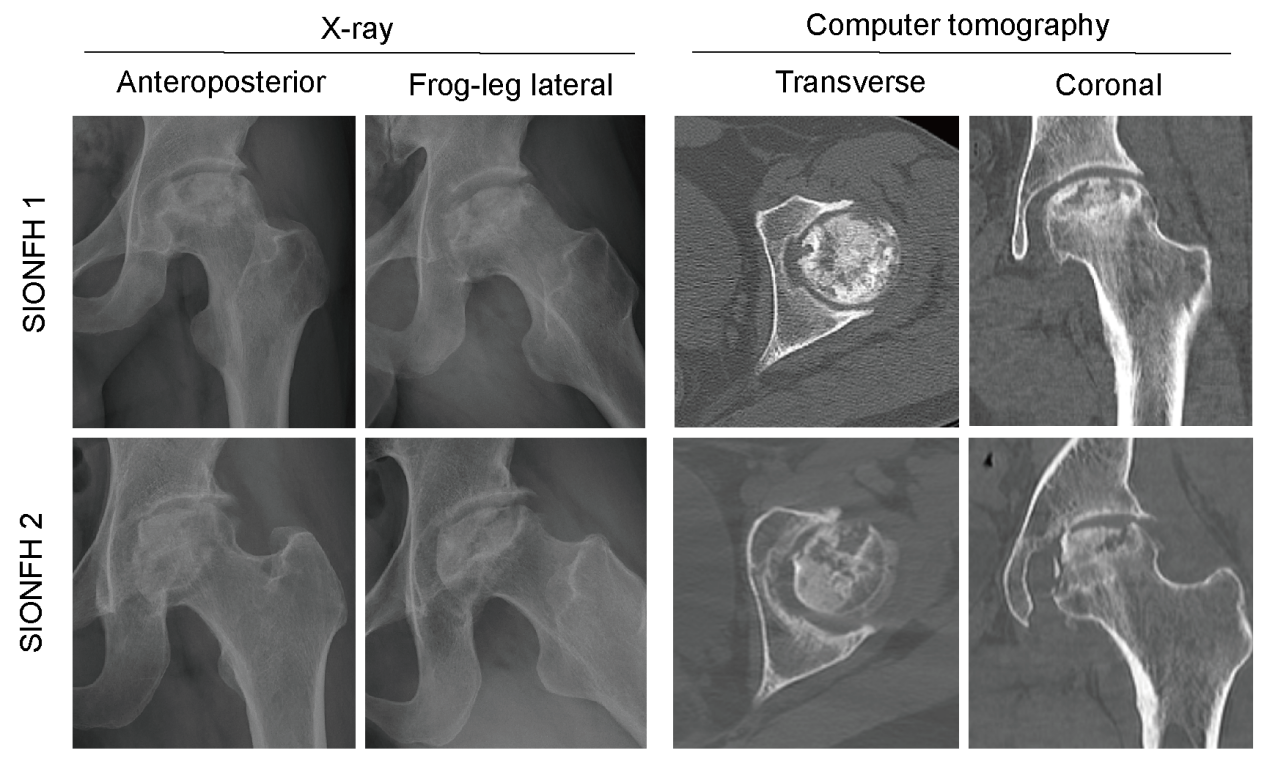


**Supplementary Figure 1.** Representative preoperative radiographs and computer tomography of patients with SIONFH undergoing total hip arthroplasty.

**Fig. S2.**


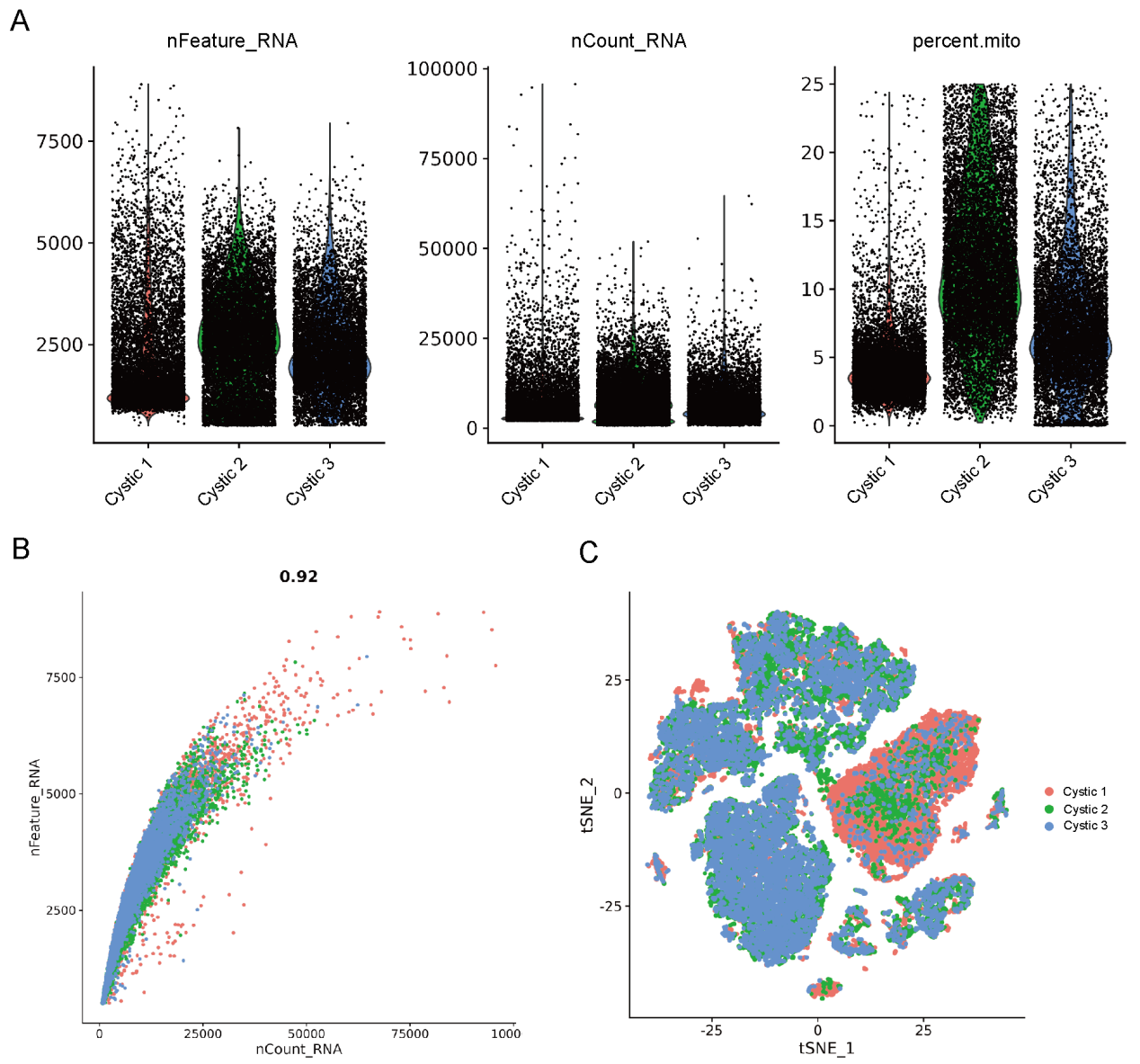


**Supplementary Figure 2.** Quality control for scRNA-seq datasets. (A) Violin plots showing the number of RNA genes, counts , and percent of mitochondrial genes in each sample. (B) The correlation between RNA counts and genes, RNA counts and mitochondrial genes in cells. (C) t-SNE plots of cells colored according to the samples. t-SNE, t-distributed stochastic neighbour embedding.

**Fig. S3.**


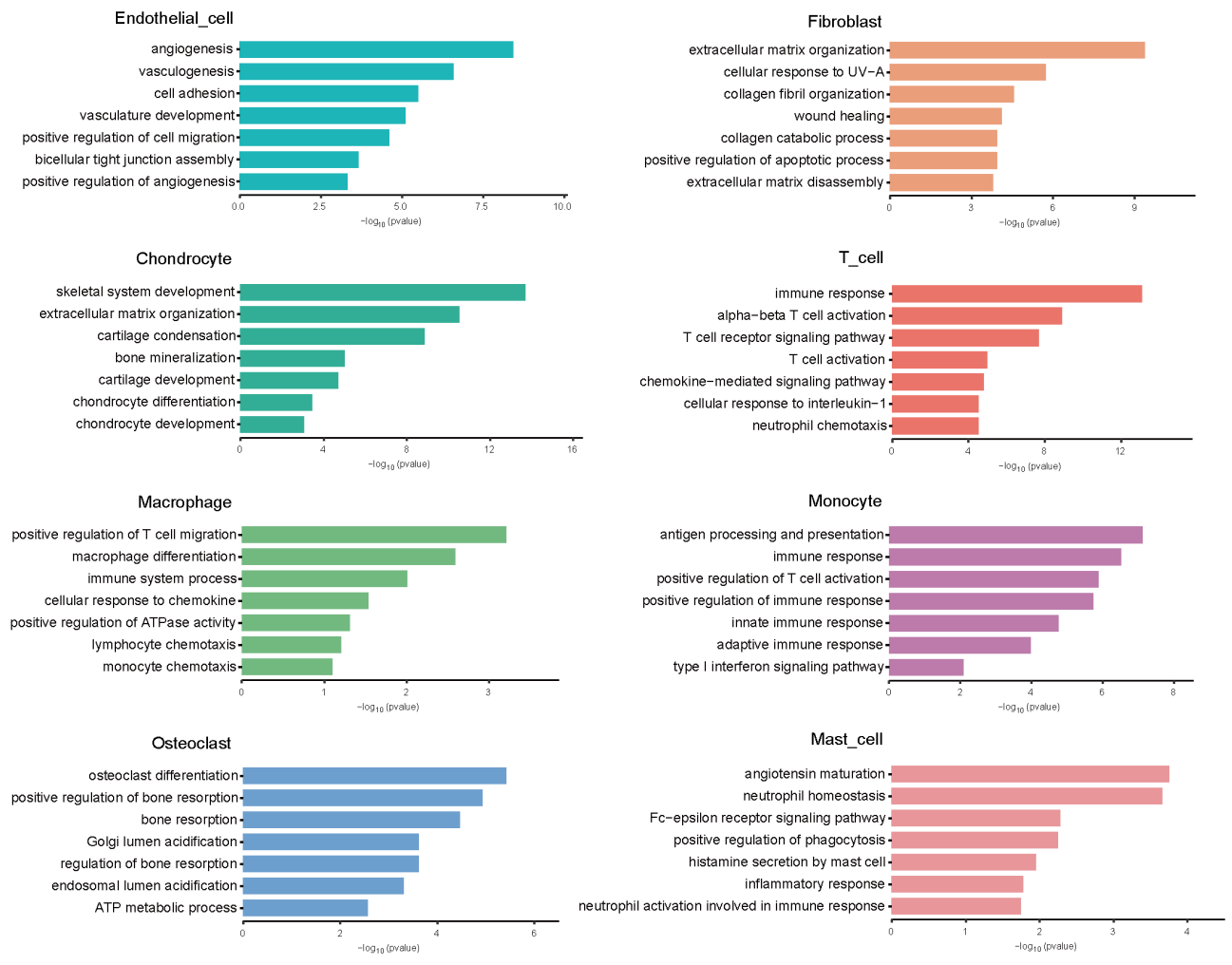


**Supplementary Figure 3.** The enriched GO terms results of marker genes of each cell subcluster. GO, gene ontology.

**Fig. S4.**


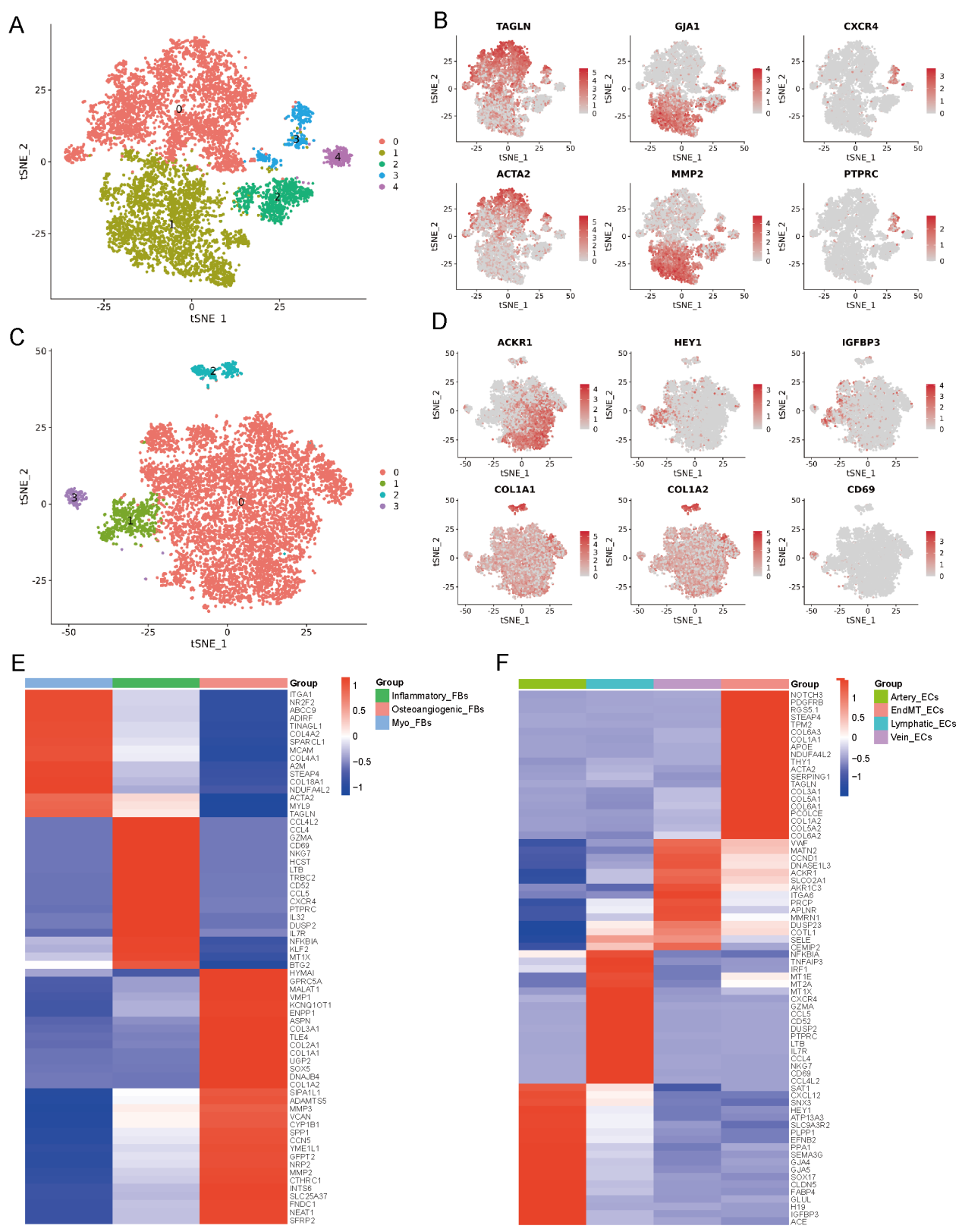


**Supplementary Figure 4.** Identification of FBs and ECs. (A) t-SNE plot showing subclusters of FBs. (B) t-SNE plots showing three major cell types of FBs identified by marker gene expression (TAGLN and ACTA2: Myo_FBs; GJA1 and MMP2: Osteogenic_FBs; CXCR4 and PTPRC: Inflammatory_FBs). (C) t-SNE plot showing subclusters of ECs. (D) t-SNE plots showing three major cell types of ECs identified by marker gene expression (ACKR1 and ACTA2: Vein_ECs; HEY1 and IGFBP3: Artery_ECs; COL1A1 and COL1A2: EndMT_FBs; CD69: Lymphatic_ECs). (E) Heatmap plot showing subclusters of FBs. (F) Heatmap plot showing subclusters of ECs. FBs, fibroblasts cells; ECs, endothelial cells; Myo_FBs,  myofibroblast cells; Osteogenic_FBs, osteogenic fibroblasts; Inflammatory_FBs, inflammatory fibroblast cells; Vein_ECs, vein endothelial cells; artery_ECs, artery endothelial cells; EndMT_ECs, endothelial mesenchymal transition endothelial cells; Lymphatic_ECs, lymphatic endothelial cells; t-SNE, t-distributed stochastic neighbour embedding.

**Fig. S5.**

**
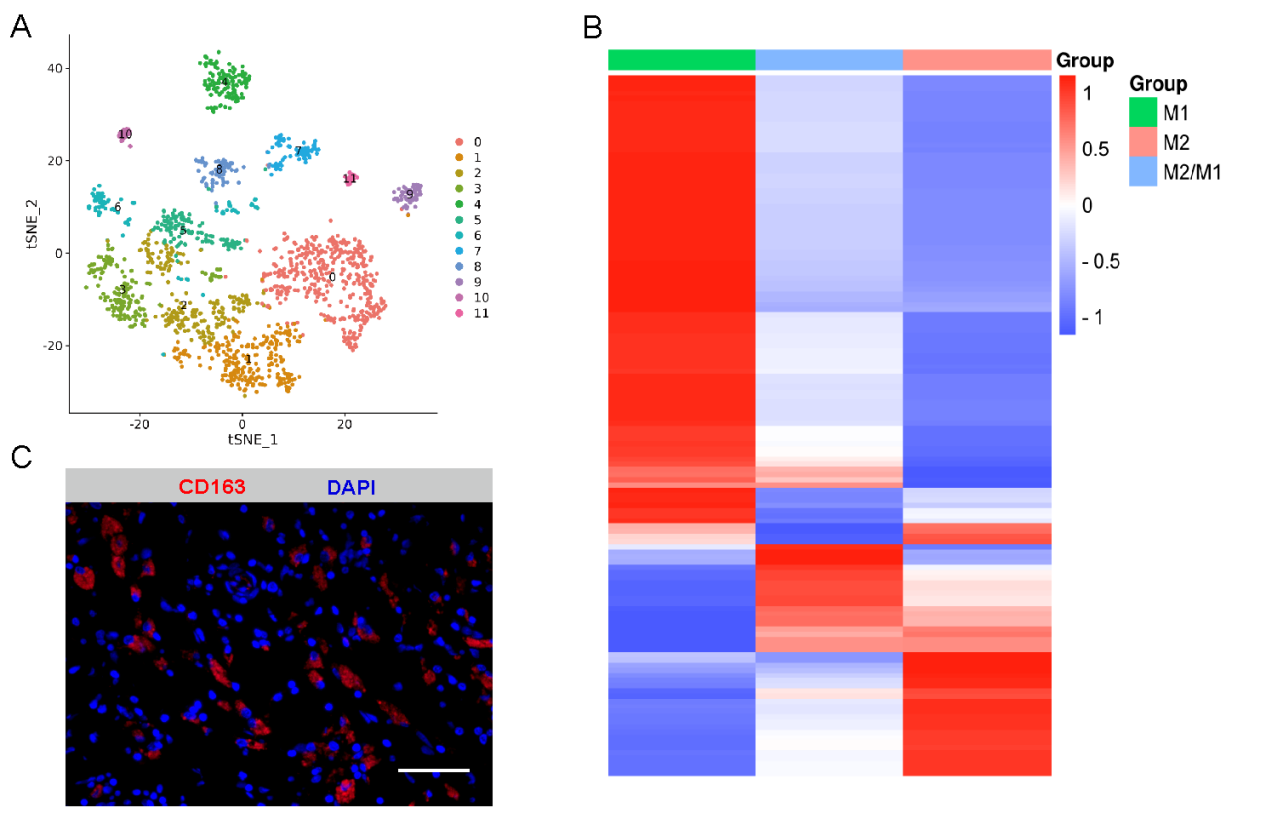
**

**Supplementary Figure 5.** Identification of macrophage cells. (A) t-SNE plot showing subclusters of macrophage cells. (B) Heatmap plot showing subclusters of macrophage cells. (C) Immunofluorescence expression of marker genes of macrophage cells. Scale bar, 50 µm. t-SNE, t-distributed stochastic neighbour embedding; M1, M1 macrophages; M2, M2 macrophages; M2/M1, M2/M1 macrophages.

**Fig. S6.**


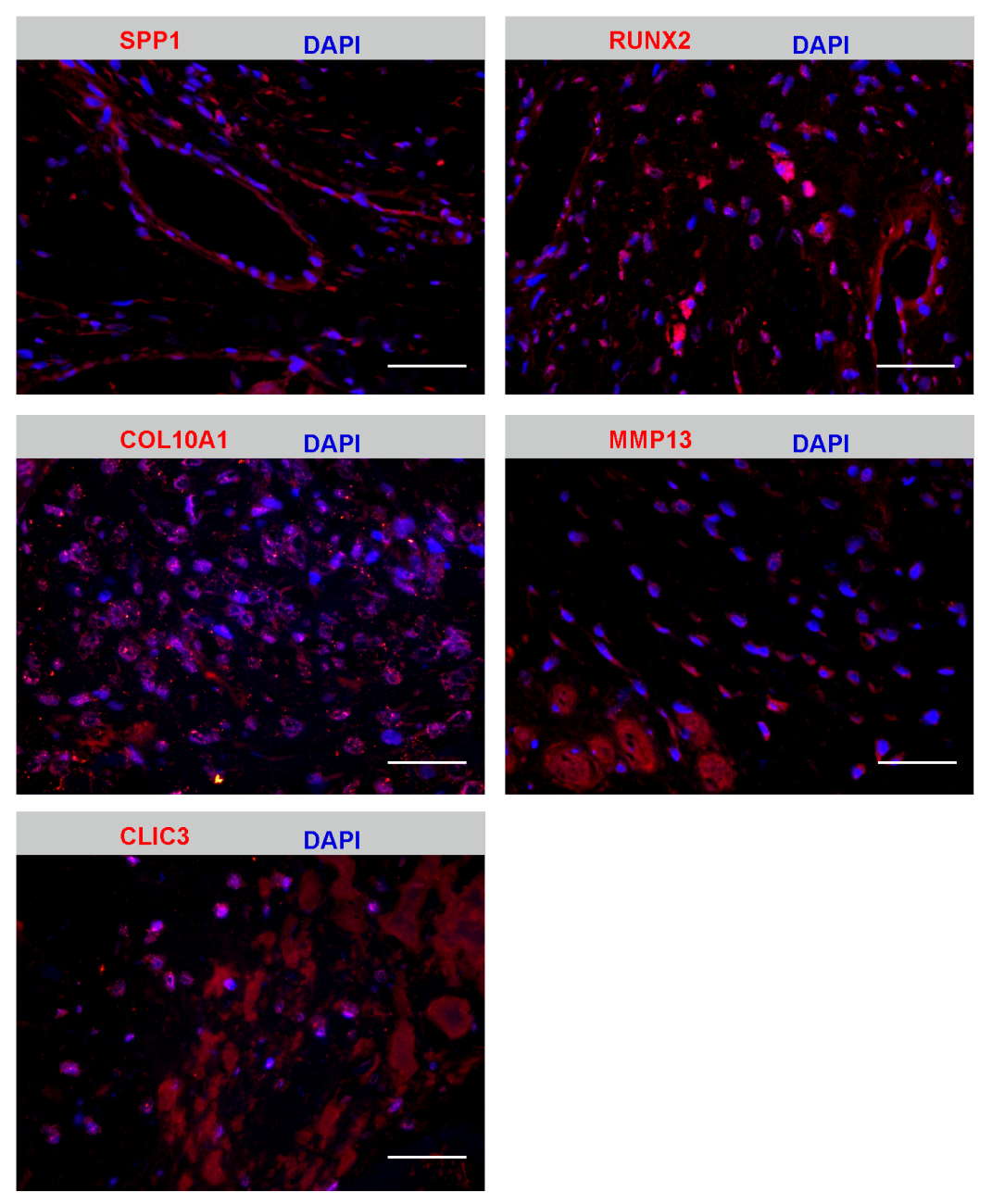


**Supplementary Figure 6.** Immunofluorescence expression of marker genes. Scale bar, 50 µm.
